# The Obesogenic and Glycemic Effect of Bariatric Surgery in a Family with a Melanocortin 4 Receptor Loss-of-Function Mutation

**DOI:** 10.3390/metabo12050430

**Published:** 2022-05-11

**Authors:** Ronit Grinbaum, Nahum Beglaibter, Stella Mitrani-Rosenbaum, Lee M. Kaplan, Danny Ben-Zvi

**Affiliations:** 1 Department of Surgery, Hadassah University Hospital Mount Scopus and the Hebrew University-Hadassah Medical School, Jerusalem 91240, Israel; bnahum@hadassah.org.il; 2 Goldyne Savad Institute of Gene Therapy, Hebrew University-Hadassah Medical School, Jerusalem 91120, Israel; stella@mail.huji.ac.il; 3 Obesity, Metabolism and Nutrition Institute, Massachusetts General Hospital, Boston, MA 02114, USA; kaplan.lee@mgh.harvard.edu; 4 Department of Developmental Biology and Cancer Research, Institute of Medical Research Israel Canada, Hebrew University-Hadassah Medical School, Jerusalem 91120, Israel

**Keywords:** obesity, bariatric surgery, diabetes, OSA, melanocortin 4 receptor

## Abstract

We report the long-term response to bariatric surgery in a singular family of four adolescents with severe obesity (41–82 kg/m^2^), homozygous for the C271R loss-of-function mutation in the melanocortin 4 receptor (MC4R), and three adults heterozygous for the same mutation. All patients had similar sociodemographic backgrounds and were followed for an average of 7 years. Three of the four homozygous patients regained their full weight (42–77 kg/m^2^), while the fourth lost weight but remained obese with a body mass index of 60 kg/m^2^. Weight regain was associated with relapse of most comorbidities, yet hyperglycemia did not relapse or was delayed. A1c levels were reduced in homozygous and heterozygous patients. The long-term follow-up data on this very unique genetic setting show that weight loss and amelioration of obesity following bariatric surgery require active MC4R signaling, while the improvement in glycemia is in part independent of weight loss. The study validates animal models and demonstrates the importance of biological signaling in the regulation of weight, even after bariatric surgery.

## 1. Introduction

Obesity is a multifactorial disease, resulting from the interaction of genetic and environmental factors. Melanocortin 4 receptor (MC4R) is a G-coupled protein receptor that is central for energy homeostasis. MC4R is expressed in neurons of the paraventricular hypothalamus that receive inputs from agouti-related protein- and proopiomelanocortin-expressing neurons that are regulated in turn by hormones such as leptin, ghrelin, and Glp1 and other metabolic and neuronal signals. MC4R signaling is, thus, central to the integration of orexigenic and anorexigenic signals and controls energy balance in mammals [1,2,3,4,5,6]. Polymorphisms in MC4R have been linked to childhood obesity and variation in body mass index (BMI) through genetic studies, and deficiency in MC4R signaling is a common form of monogenic obesity, present in up to 5% of adolescent obese patients [7,8,9,10]. Patients homozygous for a mutation leading to full deficiency in MC4R signaling are rare. These patients suffer from extreme childhood obesity and severe metabolic disorders.

Bariatric surgeries are aggressive yet currently the most effective medical treatment for obesity and several of its associated comorbidities including type 2 diabetes mellitus (T2D). The surgery leads to improvement in glycemic control and drastic weight loss, which are maintained years after surgery in many cases [11,12,13,14,15,16]. The long-term improvement in glycemic control is hypothesized to have also a weight loss-independent component attributed to an increase in Glp1 secretion, an elevation in intestinal glucose utilization, a change in the composition of the microbiome and bile acids, improved peripheral insulin sensitivity, and other mechanisms [17,18,19,20,21,22,23].

Partial or complete weight regain is not uncommon after bariatric surgery, and this regain has been linked again to polymorphisms in MC4R through genetic studies [24,25]. Other studies have shown that obese patients with partial loss of MC4R activity respond normally to surgery in terms of weight loss and glycemic control [26,27,28]. Most studies in rodent models of bariatric surgery concluded that MC4R/leptin signaling is required for weight loss after bariatric surgery, but not for improved glycemic control [23,27,29]. Altogether, these findings suggest that the mechanisms of action of bariatric surgery leading to sustained weight loss involve MC4R and the physiological pathways that regulate energy balance. However, there are few and conflicting reports on the long-term outcomes of bariatric surgery in patients with complete loss of MC4R signaling [30,31,32,33,34,35] that can provide data on the role of MC4R signaling in sustaining weight loss, and the importance of weight loss in the improvement in glycemic control following surgery in patients.

Here, we present our long-term (median 75 months) experience of the outcomes of bariatric surgeries with a family of four homozygous and three heterozygous patients carrying a C271R mutation in MC4R, leading to a disruption of disulfide bonds and a complete loss of MC4R signaling [7,36]. To our knowledge, this is the only study of such a family, with similar ethnic, genetic, environmental, and social backgrounds. Surgeries led to a transient improvement in obesity and related comorbidities in homozygous patients. Weight regain resulted in a relapse of most comorbidities. Improved glycemia was sustained years after regain, and new cases of hyperglycemia were not observed despite weight regain and obesity.

Clinically, it provides evidence that bariatric surgeries are effective for obese patients with partial loss of MC4R function. Lastly, bariatric surgeries offer much-needed transient relief from severe obesity and its comorbidities and improve long-term glycemic control even in patients suffering from severe monogenic obesity.

Our study supports the results of animal models, showing that intact MC4R signaling is critical for bariatric-surgery induced weight-loss but not for improvement in glycemic control.

## 2. Materials and Methods

**Figure 1:**
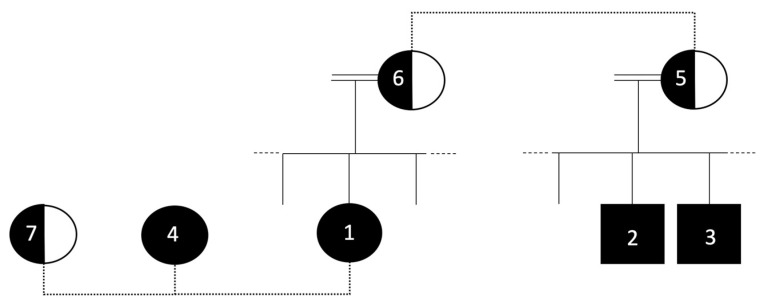
Pedigree of the patients in the study. The numbers indicate the patient number. Dashed lines denote that patients are cousins. Black circles denote homozygous females, black squares denote homozygous males, and half-filled circles denote heterozygous females. The genotype of the spouse of patients 5 and 6 is not known, but the marriage is consanguineous.

Study population: Seven patients, members of a single family with similar sociodemographic backgrounds, were treated at the same medical center since 2005. Patients 1–4 are adolescents homozygous for a C271R mutation in the *MC4R* gene, which causes a complete loss of function [7]. Patients 5–7 are adult women heterozygous for the mutation. Patients 1–4 and 7 are cousins. Patients 5 and 6 are cousins and are the mothers of patients 1 and 2–3, respectively (Figure 1). Inclusion criteria were having the C271R mutation, and having bariatric surgery. The study was conducted according to the guidelines of the Declaration of Helsinki and approved by the Ethics Committee of Hadassah Medical Center HMO 0656-21.

Clinical data: Data on glycemia, weight, height, and morbidities were retrieved retrospectively from electronic medical records. The anthropomorphic and genetic parameters of the patients before primary bariatric surgery are given in Table 1. 

Genetic analysis: DNA was extracted from blood samples, and PCR was performed using the amplification-refractory mutation system (ARMS) as described previously [37].

Statistical analysis: A paired Wilcoxon test was used to compare body mass index (BMI) and A1c levels pre-surgery and at the most recent follow-up. For patient 2, we used data before the revisional surgery as there was only a short follow-up period on this patient.

## 3. Results

### 3.1. Outcomes of Bariatric Surgery in a Family Carrying the C271R Mutation in MC4R

**Figure 2:**
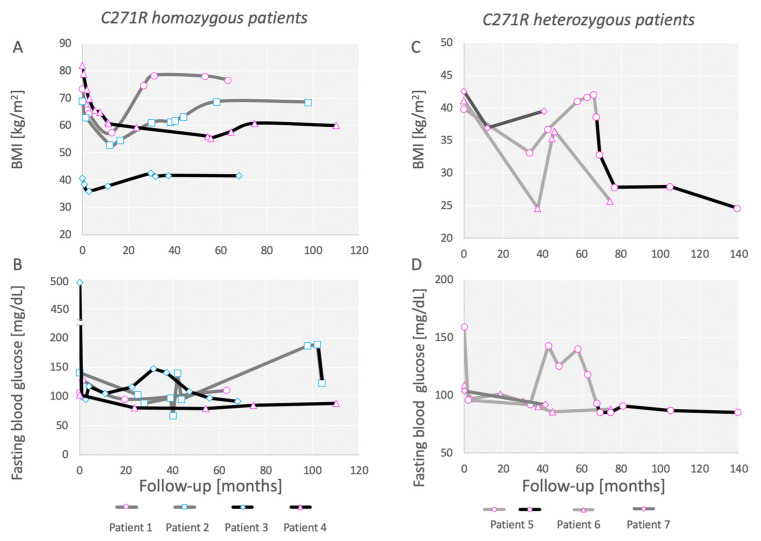
BMI and fasting blood glucose levels of patients presented in this study. (**A**,**B**) Patients carrying homozygous C271R mutation. BMI (**A**) and fasting blood glucose (**B**) as a function of time after surgery in months. Note, in (**B**), that the axis is broken over 250 mg/dL. (**C**,**D**) Patients carrying a single allele of the C271R mutation. BMI (**C**) and glucose (**D**) as a function of time after surgery in months. Color code: LAGB, light gray; LSG, dark gray; LRYGB, black. Patient 3 had a revision into LOAGB and patient 5 had a revision into LRYGB. Female patients are denoted in pink markers, while male patients are denoted in light blue.

Although individual outcomes varied, bariatric surgery did not result in a significant decrease in BMI in this small cohort (median 41.25 kg/m^2^ to 39.5 kg/m^2^, at last follow-up; *p* > 0.05). However, surgery resulted in a significant decrease in A1c (median 6.15% to 5.05%, at last follow-up; *p* < 0.05). The results are summarized in Table 2 and Figure 2.

### 3.2. Patients Homozygous for C271R Mutation in MC4R

The clinical parameters of the patients before surgery and at the most recent visit are summarized in Table 1. Patients 1–4, homozygous for the C271R MC4R mutation, presented with severe obesity and multiple debilitating comorbidities in adolescence (Table 1) and underwent bariatric surgery at ages 12–17.

Patient 1 was treated in our institute since the age of 7 for severe obstructive sleep apnea (OSA) and moderate pulmonary hypertension. She was admitted at age 11 with obstructive jaundice and diagnosed with cholelithiasis and choledocholithiasis. Endoscopic retrograde cholangial pancreatography (ERCP) was normal but was complicated afterward with post-ERCP pancreatitis. Her other comorbidities at that time were impaired fasting blood glucose, iron deficiency anemia, asthma, and tibia vara. She was on home oxygen treatment and bilevel positive airway pressure (BIPAP); she was unable to perform activities of daily living without help from her parents due to her obesity, and she was mobilized by a wheelchair. She underwent laparoscopic sleeve gastrectomy (LSG) with laparoscopic cholecystectomy with a BMI of 73.6 kg/m^2^ at the age of 12. She lost 16 kg/m^2^ in the first year and her blood CO_2_ and bicarbonate normalized. She was able to walk and partially care for herself. However, after 63 months of follow-up, she had an overall increase in BMI of 3.7 kg/m^2^, and she was back on home oxygen and BIPAP with hemoglobin of 10.4 g/dL. An abdominal CT demonstrated a narrow sleeve. She did not develop hyperglycemia, and her A1c decreased from 5.8% to 5.1%. She died of COVID-19 infection at the age of 19, 88 months after surgery.

Patient 2 is the cousin of patient 1. He had severe obesity (BMI 69 kg/m^2^), OSA treated with BIPAP, hypertension, tibia vara, and hyperglycemia (fasting blood glucose (FBG) 141 mg/dL; A1c 6.9%) at the age of 12. He had LSG, and his BMI decreased to a nadir of 53 kg/m^2^, but rose to 64 kg/m^2^ and 69 kg/m^2^ after 3.5 and 6 years, respectively. OSA and hyperglycemia reappeared (A1c 6.8%) 8 years after surgery at the age of 20, at which time he underwent an uneventful revisional surgery to laparoscopic one anastomosis gastric bypass (LOAGB). His glycemia decreased to 123 mg/dL and he was lost to follow-up.

Patient 3, who smoked dozens of cigarettes a day, is the brother of patient 2. He experienced minimal weight loss after laparoscopic Roux-en-Y gastric bypass (LRYGB) surgery at the age of 17 (BMI: 41 kg/m^2^ to 36 kg/m^2^) and regained all weight after 2.5 years. Before surgery, he had uncontrolled diabetes with an FBG of 495 mg/dL and an A1c of 12.9% treated with insulin and metformin. He also presented with hypertension, hyperlipidemia, and tibia vara which bound him to a wheelchair. Following surgery, he remained normoglycemic with A1c < 6.5% and did not require insulin in the 68 month follow-up period.

Patient 4 is a cousin of patient 1. She underwent LRYGB when she was 16. Her BMI decreased from 82 kg/m^2^ to 59 kg/m^2^ and 56 kg/m^2^ at 2.5 and 5 years after surgery, but increased to 60 kg/m^2^ after 9 years. She was normoglycemic during the entire study but suffered from anemia (Hb 9.1 g/dL) treated with IV iron. A1c levels were not measured.

### 3.3. Patients Heterozygous for C271R Mutation in MC4R

The three female heterozygous adult family members had obesity (BMI 40–43 kg/m^2^), but their disease and comorbidities were less severe compared to their homozygous relatives. Patient 5 is the mother of patients 2 and 3. She underwent laparoscopic adjustable gastric banding (LAGB) with a BMI of 40 kg/m^2^ and mild asthma, and she regained lost weight. Her hyperglycemia initially normalized but relapsed after 6 years concomitant with weight regain (A1c 6.5% to 6.2%), and she developed anemia (Hb 9.4 g/dL). She had two successful pregnancies during this period. A revisional surgery to LRYGB resulted in sustained weight loss and normoglycemia which was maintained during the 6 years of follow-up (A1c 6.2% to 5.0%). She requires IV iron therapy for the treatment of anemia.

Patient 6 is the mother of patient 1. She was treated with LAGB at the age of 41 with a BMI of 41 kg/m^2^ FBG of 109 mg/dL and mild asthma. Her BMI decreased to 26 kg/m^2^ 6 years after surgery and her FBG normalized to 88 mg/dL.

Patient 7 is a cousin of patient 4. She underwent LSG having a BMI of 43 kg/m^2^, FBG of 104 mg/dL, and OSA. She regained almost all lost weight 3.5 years after surgery (BMI 39.5 kg/m^2^), but her FGB remains at 92 mg/dL.

## 4. Discussion

**Figure 3:**
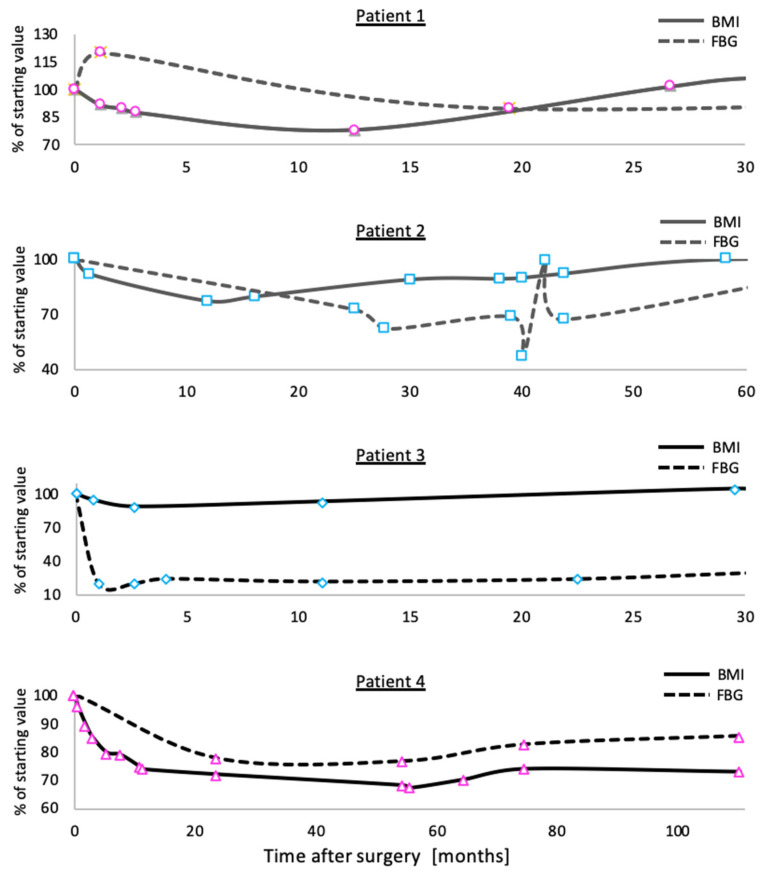
Percentage of BMI (full lines) and fasting blood glucose (FBG, dashed lines) in homozygous patients presented in this study from surgery to full weight regain (**patients 1**–**3**). **Patient 4** did not regain weight. **Patients 2**,**3** had hyperglycemia before surgery. Color coded as in Figure 2: LSG, dark gray; LRYGB, black. Female patients are denoted in pink markers, while male patients are denoted in light blue.

Three of the four homozygous patients displayed a complete regain of weight 2–5 years after surgery. The patient that sustained weight loss had a BMI of 60 kg/m^2^ 9 years after surgery and remained extremely obese. Type 2 diabetes was ameliorated in the two patients that had the disease before surgery but relapsed in one patient 8 years after surgery. It is too early to determine whether LOAGB will have a long-term effect on patient 2. Hyperglycemia did not develop in the other two patients despite the persistence of obesity (Figure 3).

Our findings confirm that patients with severe obesity associated with homozygous MC4R gene mutations show a poor sustained weight loss response to bariatric surgery [30,31,33], while heterozygous patients can experience sustained weight loss [26,27,34]. The beneficial effect of surgery on glycemic control supports previous studies showing both weight loss-dependent and weight loss-independent mechanisms resulting in improved glycemic control following bariatric surgery [38], and that sustained weight loss after surgery requires the activation of the neurological circuits controlling energy homeostasis [27].

Our findings also support conclusions in animal studies involving severe genetic obesity such as the db/db mouse showing the importance of the MC4R system in the regulation of weight following bariatric surgery, as well as the existence of weight loss-independent effects of surgery on glycemia [23,27].

Improvement in glycemic control appears to be partially independent of the MC4R signaling pathway, as A1c levels were reduced in all patients, and only one homozygous patient had a relapse of type 2 diabetes during the follow-up period. The glycemic effect is likely greater than reported since some patients reduced their use of antidiabetic drugs, and deterioration of glycemic control is expected with age. No new onset of hyperglycemia was observed. Several weight loss-independent mechanisms contributing to improved glycemia following bariatric surgery were proposed. An increase in the post-prandial secretion of glucagon-like peptide-1 (Glp1) contributes to insulin secretion [39]. Activation of the bile acid receptor FXR is important in mediating the effects of sleeve gastrectomy [40]. Bile acid concentration and composition in plasma change after surgery, possibly due to changes in the bile microbiome [18]. Other studies recorded an increase in hepatic insulin sensitivity and a reduction in hepatic gluconeogenesis following bariatric surgeries [23,38]. Drugs that may reconstitute some of the effects of surgery, e.g., Glp1 agonists and FXR agonists, or fecal transplant from patients that underwent a successful surgery may also lead to new treatments for lean patients that live with type 2 diabetes or in the prediabetes phase.

Patient 6 underwent LAGB, patients 1, 2, and 7 had LSG, and patients 3 and 4 had LRYGB. Patient 5 had LRYGB following a failed LAGB. The different procedures have different metabolic effects on, e.g., secretion of Glp1 or change in the gut microbiome [41,42,43]. It is possible that the rapid regain of weight observed in patients 1 and 2 but not in patient 3 can be explained by the differences between LSG and LRYGB. Relapse of fasting hyperglycemia in patient 2 but not in patient 4 can also be attributed to the stronger effect of LRYGB on the secretion of incretins.

The study is limited by the small number of patients and the similarity between patients, which caution against generalizing the results. On the other hand, the variability between patients in terms of age, sex, comorbidities, compliance to medical treatment (which was generally poor), the type of bariatric surgery, and variability of the follow-up period suggest that several confounding factors limit the interpretation of the data. For example, patients 1–3 suffered from tibia vara, which limited their mobility, and patient 3 smoked over 30 cigarettes a day during most of the time he was treated. This study is also limited by the lack of information on metabolic rate, dietary habits, and levels of hormones such as insulin and leptin.

A strength of this long-term study is that all patients are members of the same family and of similar sociodemographic backgrounds, and they were treated in the same medical center. This is, to our knowledge, the largest family with such a mutation that was treated by bariatric surgery. Beyond clinical experience, it provides mechanistic support for animal studies on genetic models for obesity on mechanisms driving weight loss and improvement in glycemia following bariatric surgery [23,27].

## 5. Conclusions

In adolescent patients with MC4R-related obesity, the decision to recommend bariatric surgery should be made with caution, as it may lead to long-term deleterious effects. In addition to surgical risks, it can cause adverse consequences for bone metabolism and lead to malabsorptive disorders, especially in patients with low compliance to follow-up management [44]. Although bariatric surgery appears to be ineffective in the long-term treatment of obesity in these patients, it may have long-term benefits for patients with comorbidities such as hyperglycemia. Furthermore, even if transient, weight loss may have beneficial developmental and psychological effects on the adolescent. The decision of whether to recommend bariatric surgery to adolescent patients with genetic obesity is complex and requires a multidisciplinary, holistic approach.

## Figures and Tables

**Table 1:** Anthropomorphic and genetic parameters of the patients before primary bariatric surgery. LAGB: laparoscopic adjustable gastric banding; LSG: laparoscopic sleeve gastrectomy; LRYGB: laparoscopic Roux-en-Y gastric bypass.

#	Year	Age	Sex	Weight (kg)	Height (cm)	Surgery	C271 Mutation
1	2013	12	F	159	147	LSG	Hom
2	2011	12	M	167	156	LSG	Hom
3	2010	17	M	111	165	LRYGB	Hom
4	2005	16	F	200	157	LRYGB	Hom
5	2007	35	F	102	167	LAGB	Het
6	2010	41	F	99	155	LAGB	Het
7	2016	18	F	109	160	LSG	Het

**Table 2:** Summary of cases. The four adolescent patients homozygous for the C271R mutation in MC4R are in the top rows (Hom), and the three heterozygous patients are below (Het). ^§^ Values are follow-up months since revisional surgery. LAGB: laparoscopic adjustable gastric banding; LSG: laparoscopic sleeve gastrectomy; LRYGB: laparoscopic Roux-en-Y gastric bypass; LOAGB: laparoscopic one anastomosis gastric bypass; BMI: body mass index; A1c: glycated hemoglobin; OSA: obstructive sleep apnea; T2D: type 2 diabetes mellitus; IFG: impaired fasting glucose. Glucose levels reflect fasting plasma glucose.

	General				Main Clinical Parameters at the Surgery					Main Clinical Parameters at the Latest Time Point					
*#*	*C271R Mutation*	*Sex*	*Bariatric Surgery*	*Year, Age at Surgery*	*BMI (kg/m^2^)*	*Glucose (mg/dL)*	*A1c (%)*	*Blood Pressure (mmHg)*	*Major Morbidities*	*Followup (Months)*	*BMI (kg/m^2^)*	*Glucose (mg/dL)*	*A1c (%)*	*Blood Pressure (mmHg)*	*Major Morbidities*
1	Hom	F	LSG	2013, 12	73.6	106	5.8	149/118	Anemia, asthma, OSA, hypertension, tibia vara	63	76.7	110	5.1	123/72	OSA, asthma, tibia vara
2	Hom	M	LSG	2012, 12	68.9	141	6.9	141/113	T2D, OSA, hypertension, tibia vara	98	68.6	189	6.8	128/65	T2D, OSA, tibia vara
			LOAGB	2020, 20	68.6	189	6.8	128/65	T2D, OSA, tibia vara	1 ^§^	67.5	123	-	-	OSA, tibia vara
3	Hom	M	LRYGB	2010, 17	40.8	495	12.9	139/78	T2D, hypertension, hyperlipidemia, tibia vara	68	41.7	92	6.2	133/72	Tibia vara
4	Hom	F	LRYGB	2005, 16	82.2	76	-	140/105	Hypertension	110	60	88	-	142/80	Anemia, hypertension
5	Het	F	LAGB	2007, 35	39.8	159	6.5	125/73	T2D, asthma	67	38.7	93	6.2	138/87	Hypertension
5			LRYGB	2013, 41	38.7	93	6.2	138/87	Hypertension	72 ^§^	24.6	85	5	141/87	Hypertension, anemia
6	Het	F	LAGB	2010, 41	41.2	109	5.1	125/75	IFG, asthma	75	25.8	88	4.5	-	
7	Het	F	LSG	2016, 18	42.6	104	5.6	133/76	IFG	41	39.5	92	4.5	126/70	

## Data Availability

Data for this study are available upon request from the authors.
